# Structural basis of SARS-CoV-2 and its variants binding to intermediate horseshoe bat ACE2

**DOI:** 10.7150/ijbs.73640

**Published:** 2022-07-11

**Authors:** Lingfeng Tang, Di Zhang, Pu Han, Xinrui Kang, Anqi Zheng, Zepeng Xu, Xin Zhao, Vivien Ya-Fan Wang, Jianxun Qi, Qihui Wang, Kefang Liu, George F. Gao

**Affiliations:** 1 CAS Key Laboratory of Pathogen Microbiology and Immunology, Institute of Microbiology, Chinese Academy of Sciences, Beijing 100101, China; 2 Faculty of Health Sciences, University of Macau, Macau SAR 999078, China; 3 University of Chinese Academy of Sciences, Beijing 100049, China

**Keywords:** SARS-CoV-2, intermediate horseshoe bats, bACE2-Ra, structure, SARS-CoV-2 variants

## Abstract

Coronavirus disease 2019 (COVID-19), caused by severe acute respiratory syndrome coronavirus 2 (SARS-CoV-2), has caused a global pandemic. Intermediate horseshoe bats (*Rhinolophus affinis*) are hosts of RaTG13, the second most phylogenetically related viruses to SARS-CoV-2. We report the binding between intermediate horseshoe bat ACE2 (bACE2-Ra) and SARS-CoV-2 receptor-binding domain (RBD), supporting the pseudotyped SARS-CoV-2 viral infection. A 3.3 Å resolution crystal structure of the bACE2-Ra/SARS-CoV-2 RBD complex was determined. The interaction networks of Patch 1 showed differences in R34 and E35 of bACE2-Ra compared to hACE2 and big-eared horseshoe bat ACE2 (bACE2-Rm). The E35K substitution, existing in other species, significantly enhanced the binding affinity owing to its electrostatic attraction with E484 of SARS-CoV-2 RBD. Furthermore, bACE2-Ra showed extensive support for the SARS-CoV-2 variants. These results broaden our knowledge of the ACE2/RBD interaction mechanism and emphasize the importance of continued surveillance of intermediate horseshoe bats to prevent spillover risk.

## Introduction

Bats serve as reservoir hosts of alpha and beta coronaviruses (CoVs), including severe acute respiratory syndrome coronavirus 2 (SARS-CoV-2) related CoVs 1, 2. Horseshoe bats are widely distributed in East and Southeast Asia, and SARS-CoV-2 related viral genome sequences from horseshoe bats have been reported 2-7. RaTG13, sequenced from *Rhinolophus affinis* (intermediate horseshoe bat), is the second closest relatives of SARS-CoV-2, the causative agent of global COVID-19 pandemic 8-10. The nucleotide identity between RaTG13 and SARS-CoV-2 at the whole genome scale is 96.2%, while the amino acid identity of the receptor-binding domain (RBD) is 89.3% 2. Bioinformatic analyses estimated that RaTG13 and SARS-CoV-2 diverged 40-70 years ago, most probably in horseshoe bats 11. The binding capacity of RaTG13 to humans and other species has been reported, suggesting that intermediate horseshoe bats are a potential reservoir for infectious SARS-CoV-2-related viruses 12. RaTG15, a recently reported SARS-related CoV, has also been identified in intermediate horseshoe bats 13. Considering the similarity between RaTG13 and SARS-CoV-2, the host potential of intermediate horseshoe bats must be investigated.

The virus-receptor interaction is a key factor in determining the tissue tropism and host range of CoVs, and receptor binding is critical for viral infection and transmission 14, 15. The first step of SARS‐CoV‐2 invasion is binding to the angiotensin converting enzyme 2 (ACE2) receptor on host cells via the RBD of spike (S) glycoprotein 16. SARS-CoV-2 has a broad host range, including bats 17. Recent studies have shown the susceptibility of ACE2 orthologs from 46 bat species and their ability to support the entry of SARS-CoV-2 through virus-host receptor binding and infection assays 18. However, the virus-host receptor-binding demonstrates dramatic variation among bat species. After intranasal infection in lab, oral and fecal shedding were observed on *Rousettus aegyptiacus* (Egyptian fruit bat), indicating that Egyptian fruit bats are at risk of zoonotic infection with SARS-CoV-2^19^.

Some homologous models of ACE2/RBD complexes have been determined, including those from human, cat, big-eared horseshoe bat, pangolin and dog 16, 17, 20, 21. These structures revealed different mechanisms involving hydrogen bonds and van der Waals forces based on identified critical residues in ACE2. These residues include D30, K31, Y41, Q42 and K353. Moreover, the structure of RaTG13 RBD binding to human ACE2 has been resolved, suggesting that RaTG13 from intermediate horseshoe bats presents a cross-species and potential pandemic risk 12. However, the interaction mechanism requires further study because substitutions that contribute to ACE2 diversity and RBD variants would greatly affect the affinity and host range. To better evaluate the interaction between the intermediate horseshoe bat and SARS-CoV-2, the molecular basis of ACE2 binding to the RBD should be explored.

The emergence of new SARS-CoV-2 variants, including B.1.1.7 (Alpha), B.1.351 (Beta), P.1 (Gamma), B.1.617.2 (Delta) and B.1.1.529 (Omicron), have caused global concerns owing to their significantly increased transmission and gradually superseding as the main strains. Notably, the RBD substitutions of these variants are primarily concentrated in the critical residues K417, L452, T478, E484, F490 and N501 22. Two other mutations, Q493 and Q498, were identified in the S protein in mouse-adapted strains 23-25. In addition, comparative genomics found significant signals of specific selection and accelerated evolution in the bat ACE2 lineage 26. However, whether these variants influence adaptability to intermediate horseshoe bats has not yet been investigated.

In this study, we evaluated the interaction between the SARS-CoV-2 RBD and intermediate horseshoe bat ACE2 (bACE2-Ra), and investigated the infection efficiency of pseudotyped SARS-CoV-2 via bACE2-Ra. We also revealed the molecular mechanism using structural biology and identified two residues on the interface of the SARS-CoV-2 RBD are crucial for the binding to bACE2-Ra and hACE2. Moreover, bACE2-Ra broadly recognizes SARS-CoV-2 variant RBDs. These results broadened our knowledge of the molecular mechanisms of SARS-CoV-2 RBD binding to bACE2-Ra, suggesting that long-term monitoring of intermediate horseshoe bats is vital to minimize spillover risk.

## Results

### Binding of SARS-CoV-2 RBD with bACE2-Ra and infectivity of pseudotyped SARS-CoV-2

**Figure 1:**
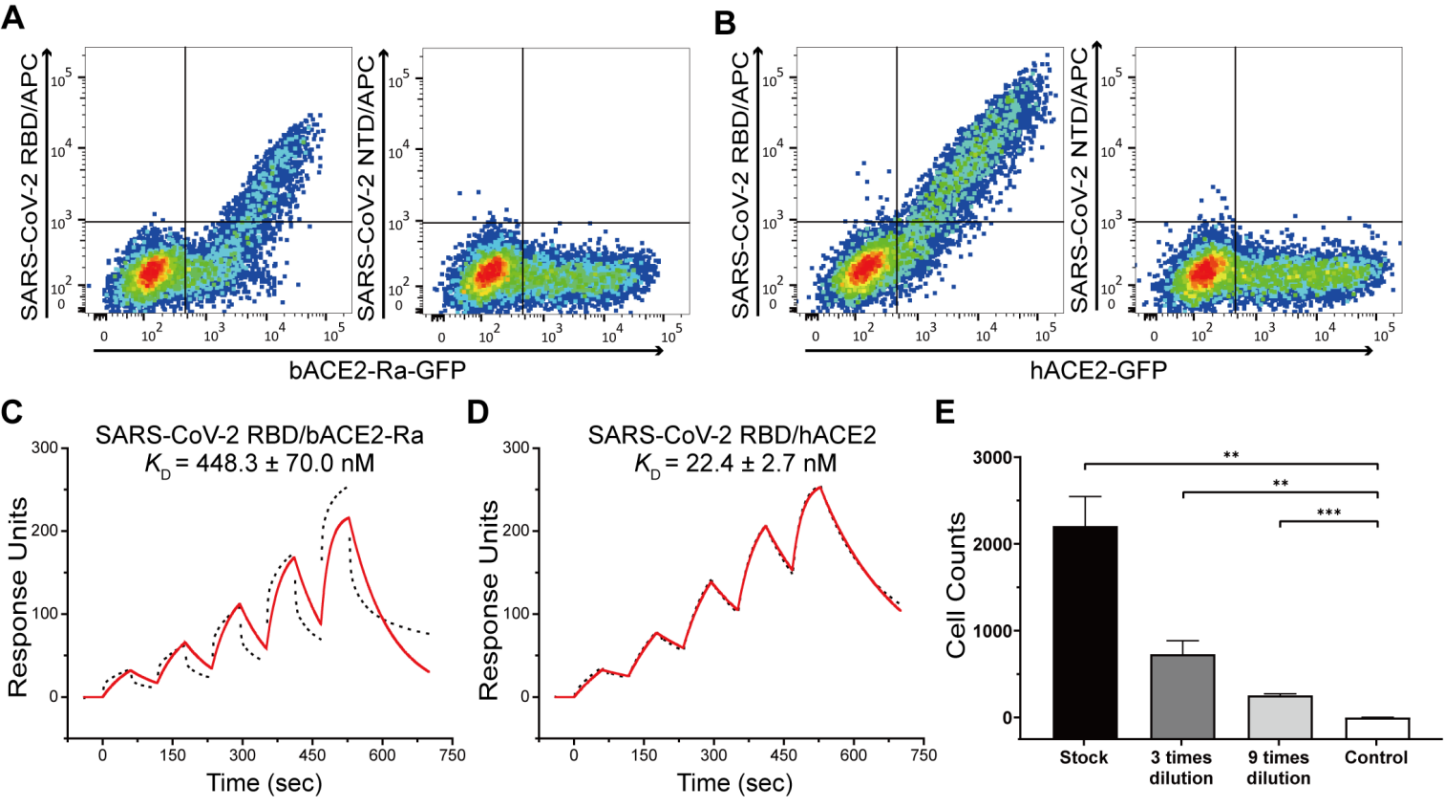
** Binding test between SARS-CoV-2 RBD and bACE2-Ra and the infection of pseudotyped SARS-CoV-2. A & B.** Flow cytometry assay of SARS-CoV-2 RBD binding to bACE2-Ra- (A) or hACE2- (B) expressing BHK cells. SARS-CoV-2 NTD was set as the negative control.** C & D.** The binding affinity between SARS-CoV-2 RBD and bACE2-Ra (C) or hACE2 (D) as determined by SPR assay. The raw and fitted curves are represented as black dotted lines and red solid lines, respectively.** E.** Pseudotyped SARS-CoV-2 infection of HeLa-bACE2-Ra cells. HeLa cells were set as the negative control. **P < 0.01, ***P < 0.001 (Student's *t*-test, two-tailed).

We first evaluated the binding capacity of the SARS-CoV-2 RBD to bACE2-Ra using flow cytometry. BHK-21 cells were transiently transfected to express bACE2-Ra-eGFP or hACE2-eGPF on the surface, and the SARS-CoV-2 S protein NTD was used as the negative control. The results showed that SARS-CoV-2 RBD could bind to bACE2-Ra-expressing BHK-21 cells. However, the double-positive rate (31.6%) was lower than that of hACE2-expressing cells (69.5%), which qualitatively indicated that the binding affinity of SARS-CoV-2 RBD to bACE2-Ra was lower than that of hACE2 **(Fig. 1A and B; Fig. S1A and B)**. Surface plasmon resonance (SPR) assay was then performed to quantitatively measure the binding affinity of bACE2-Ra to the SARS-CoV-2 RBD, with hACE2 as the positive control. The binding affinity of bACE2-Ra to the SARS-CoV-2 RBD (K_D_ = 448.3 nM) was lower than that of hACE2 (*K*_D_ = 22.4 nM), consistent with the flow cytometry results **(Fig. 1C and D)**. To investigate whether SARS-CoV-2 could infect host cells by binding to bACE2-Ra, vesicular stomatitis virus (VSV)-based pseudotyped SARS-CoV-2 was serially diluted and transduced with bACE2-Ra-expressing HeLa cells (HeLa-bACE2-Ra), and HeLa cells were designated as the negative control. The results showed that pseudotyped SARS-CoV-2 was successfully transfected into HeLa-bACE2-Ra cells **(Fig. 1E)**.

### Complex structure of SARS-CoV-2 RBD with bACE2-Ra

To further investigate the molecular basis of the interaction between bACE2-Ra and SARS-CoV-2 RBD, we prepared SARS-CoV-2 RBD in complex with bACE2-Ra **(Fig. S1C)** and resolved its crystal structure. There were four ACE2/RBD complexes in one asymmetric unit, and the resolution of the complex was determined at 3.3 Å **(Table S1)**. Overall, the architecture of bACE2-Ra with the SARS-CoV-2 RBD is similar to that of the hACE2/SARS-CoV-2 RBD complex (PDB ID: 6LZG), with a root-mean-square deviation (RMSD) of 0.733 Å for 730 atoms.

**Figure 2:**
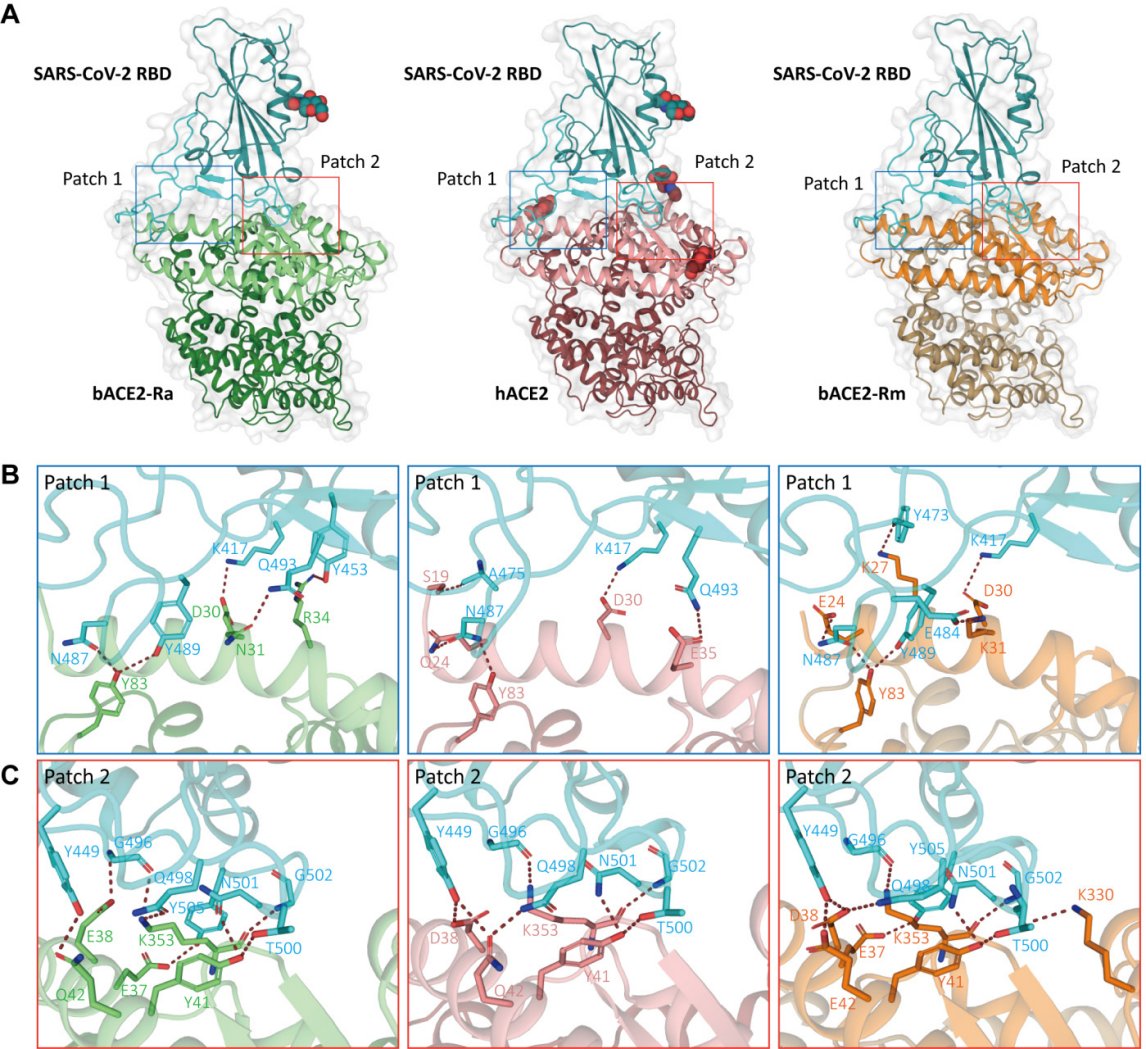
** Complex structures of SARS-CoV-2 RBD binding to bACE2-Ra, hACE2 and bACE2-Rm. A.** The overall complex structures of SARS-CoV-2 RBD (cyan) binding to bACE2-Ra (green), hACE2 (salmon) and bACE2 Rm (orange). The core and external regions are painted with light and dark colors, respectively. The binding between RBD and ACE2s, mainly composed of two patches of interactions, Patch 1 and Patch 2 respectively, are shown in detail. The N‐glycans are shown as spheres. **B & C.** Detailed interactions of RBD with bACE2-Ra, hACE2 and bACE2-Rm in Patch 1 (B) and Patch 2 (C). Critical residues are labeled, and hydrogen bonds (polar contacts within 3.5 Å) are shown as red dotted lines.

SARS-CoV-2 RBD bound to bACE2-Ra with an external subdomain composed of flexible loops between two β-sheets. The interaction surface was distributed into two patches** (Fig. 2A)**. Patch 1 mainly contained two α-helices on the N-terminus (α1 and α2), while Patch 2 included α1 and a pair of anti-parallel β-sheets (β4 and β5) of ACE2 **(Fig. 2B and C)**. We compared the interaction network with the hACE2/SARS-CoV-2 RBD and bACE2-Rm/SARS-CoV-2 RBD. In Patch 1 of bACE2-Ra, R34 formed hydrogen bonds with Y453 in the SARS-CoV-2 RBD. However, such hydrogen bonds did not exist in hACE2/SARS-CoV-2 RBD or bACE2-Rm/SARS-CoV-2 RBD complex, as the spatial distance between Y453 in the SARS-CoV-2 RBD and H34 in hACE2 (3.8 Å) or S34 in bACE2-Rm (4.2 Å) was too long to form hydrogen bonds. D30 and Y83 of bACE2-Ra interacted conservatively with K417 and N487 of RBD respectively **(Fig. 2B)**. N31 of bACE2-Ra was also responsible for binding to SARS-CoV-2 RBD, however, it interacted with Q493 instead of E484 compared to K31 of hACE2 and bACE2-Rm **(Fig. 2B)**. In Patch 2, compared to hACE2, residue E38 of bACE2-Ra formed a hydrogen bond with G496 instead of Y449 of SARS-CoV-2 RBD, whereas Q42 formed a hydrogen bond with Y449 instead of Q498. E37, Y41 and K353 of bACE2-Ra/hACE2/bACE2-Rm were relatively conservative, that they formed hydrogen bonds with G496, Q498, T500, N501, G502 and Y505 of RBD respectively **(Fig. 2C)**.

### Comparison of SARS-CoV-2 RBD binding to bACE2-Ra, hACE2 or bACE2-Rm

**Figure 3:**
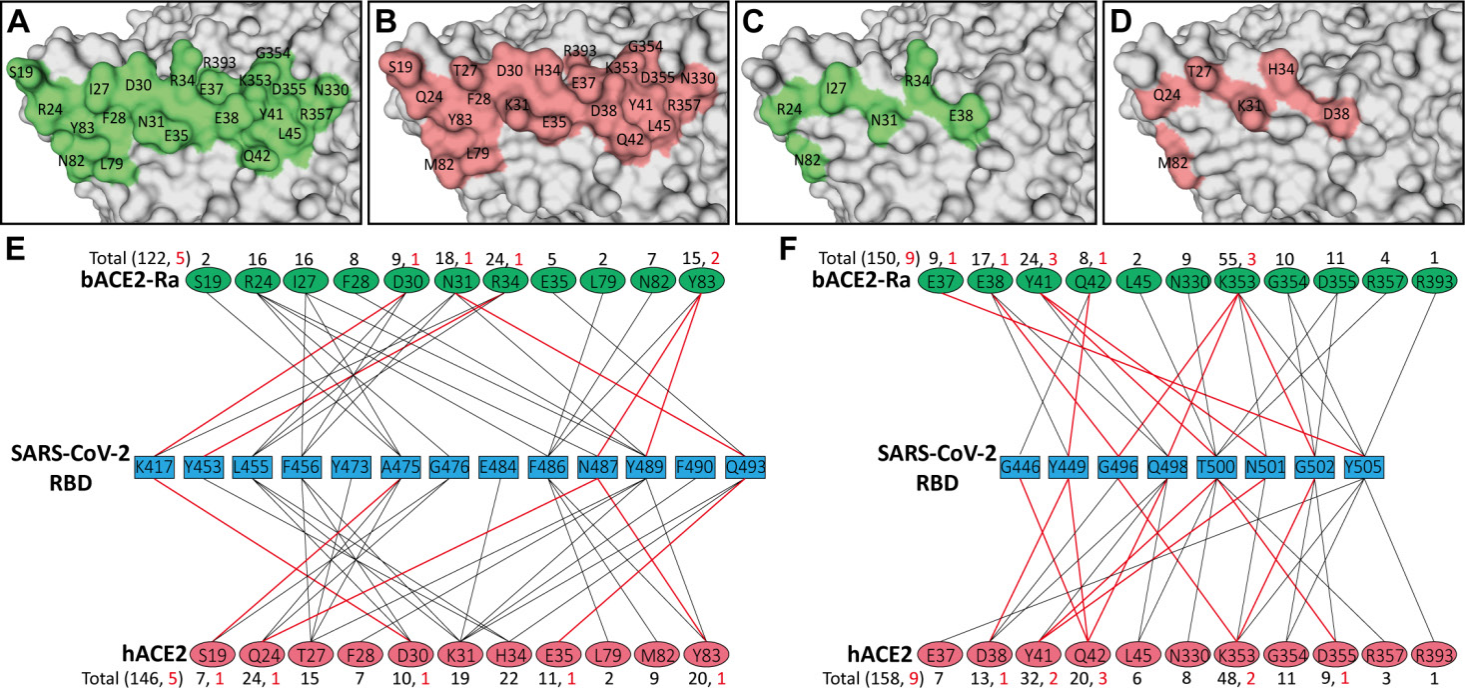
** Comparison of the binding between bACE2-Ra and hACE2, and identification of determinants between RBD and bACE2-Ra. A-D.** The interaction interface of bACE2-Ra (A) or hACE2 (B). Different interacting residues between bACE2-Ra (C) and hACE2 (D) on the interaction interface are shown apart. **E & F.** The interaction networks of SARS-CoV-2 RBD with bACE2-Ra or hACE2. Black lines indicate Van der Waals contacts (within 4.5 Å), and red lines represent hydrogen bonds or salt bridges (within 3.5 Å).

To analyze the characteristics and differences of all the residues participating in the binding process, the interfaces of bACE2-Ra, hACE2, and bACE2-Rm were displayed. Patch 1 of bACE2-Ra contains eleven residues (S19, R24, I27, F28, D30, N31, R34, E35, L79, N82 and Y83), while Patch 2 contains another eleven (E37, E38, Y41, Q42, L45, N330, K353, G354, D355, R357 and R393) **(Fig. 3A, E and F; Fig. S2A)**. Comparing with hACE2, Patch 2 was relatively conservative, with substitution only in residue 38 **(Fig. 3B-D, and F)**. For Patch 1, there were apparent differences in residues 31 and 34. N31 and R34 of bACE2-Ra formed additional hydrogen bonds with Q493 and Y453 of SARS-CoV-2 RBD, respectively. However, unlike K31 of hACE2 or bACE2-Rm, N31 of bACE2-Ra did not interact with E484 and F490 of the RBD **(Fig. 3E; Table 1)**. Additionally, bACE2-Rm contains five (E24, K27, K31, S34, K35) and three different residues (D38, E42, K330) in Patch 1 and 2, respectively, whose characteristics are different from those of bACE2-Ra, except D38 **(Fig. S2B-E)**.

### E35K substitution of bACE2-Ra enhanced the binding to SARS-CoV-2 RBD

**Figure 4:**
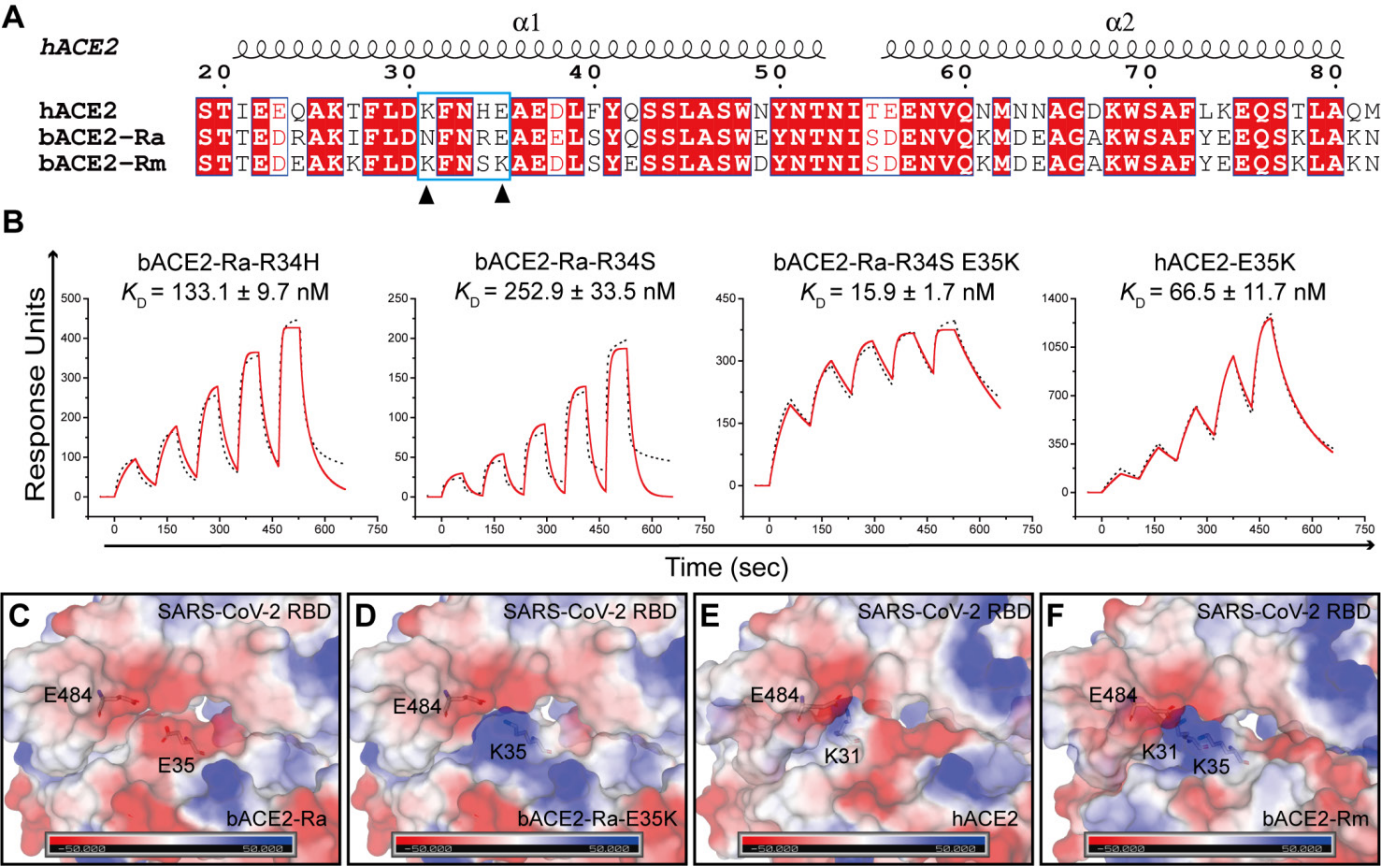
** SPR assay of the binding between bACE2-Ra mutants and SARS-CoV-2 RBD. A.** Sequence alignment of α1 and α2 helix of bACE2-Ra, hACE2 and bACE2-Rm. Residues 31 and 35 are marked with black triangles. **B.** SPR assay depicting the binding affinity between SARS-CoV-2 RBD and bACE2-Ra mutants. The raw and fitted curves are shown as black dotted lines and red solid lines, respectively.** C-F.** The surface electrostatic potential of bACE2-Ra (C), hACE2 (E), bACE2-Rm (F) and predicted bACE2-Ra-E35K (D). The scale is from -50.0 to +50.0 kTe^-1^.

Both residues 34 and 35 of bACE2-Ra played vital roles in SARS-CoV-2 RBD binding. Sequence alignment showed that residue 34 of ACE2 is diverse among humans, intermediate horseshoe bats and big-eared horseshoe bats** (Fig. 4A).** Residue 35 of both human and intermediate horseshoe bat ACE2s is negatively charged glutamic acid (E), whereas residue 35 of the big-eared horseshoe bat is a positively charged lysine (K). To investigate the role of these two residues for SARS-CoV-2 RBD binding, we substituted R34 of bACE2-Ra with H34 and S34, and compared their binding affinities with SARS-CoV-2 RBD. Compared with prototype bACE2-Ra, the binding affinity of bACE2-Ra-R34H to SARS-CoV-2 RBD increased approximately three-fold, and bACE2-Ra-R34S showed a similar binding affinity to prototype bACE2-Ra. When the E35K substitution was introduced to bACE2-Ra-R34S, the binding affinity to the SARS-CoV-2 RBD was substantially increased (~25-fold)** (Fig. 1C; Fig. 4B)**. In contrast, the E35K substitution in hACE2 decreased the binding to SARS-CoV-2 RBD (~11-fold) **(Fig. 1D; Fig. 4B)**.

As E and K are oppositely charged, we analyzed the surface electrical properties of bACE2-Ra, hACE2 and bACE2-Rm, as well as the SARS-CoV-2 RBD. In the bACE2-Ra/SARS-CoV-2 RBD complex, both E35 in bACE2-Ra and E484 in the SARS-CoV-2 RBD are negatively charged amino acids that repel each other **(Fig. 4C)**. When E35 of bACE2-Ra was substituted by the positively charged amino acid, K35, the charge on the corresponding region of bACE2-Ra changed to the positive charge to enhance the binding affinity with E484 in the SARS-CoV-2 RBD **(Fig. 4D)**. In the hACE2/SARS-CoV-2 RBD complex and bACE2-Rm/SARS-CoV-2 RBD complex, K31 of hACE2 or bACE2-Rm contacted E484 of the SARS-CoV-2 RBD, whereas E35 in hACE2 or K35 in bACE2-Rm did not **(Fig. 4E and F)**. Therefore, the E35K substitution had almost no influence on their binding affinity **(Fig. 4B)**.

### Binding capacity of SARS-CoV-2 variant RBDs to bACE2-Ra

**Figure 5:**
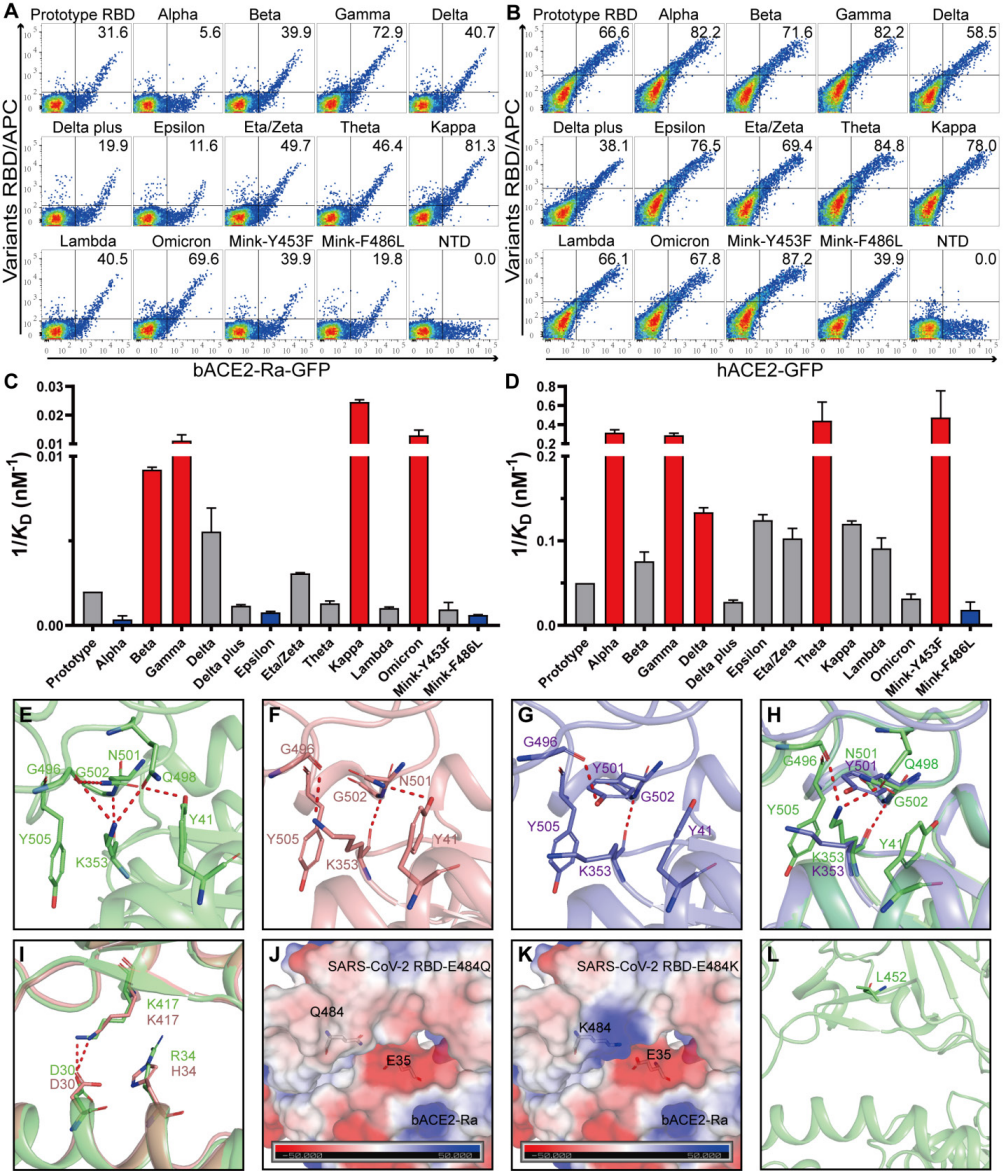
** Binding assays between bACE2-Ra and SARS-CoV-2 mutants RBD. A & B.** Flow cytometry assay of SARS-CoV-2 mutants RBD binding to bACE2-Ra- (A) or hACE2- (B) expressing BHK cells. SARS-CoV-2 NTD was set as the negative control. **C & D.** Histograms of SPR assay depicting the binding affinity between SARS-CoV-2 mutants RBD and bACE2-Ra (C) or hACE2 (D). The vertical axis denotes the 1/*K*_D_ value. Significant increases are marked in red (more than threefold), while significant attenuations are marked in bule (less than threefold).** E-H.** The interactions at the vicinity of residue N501. bACE2-Ra (E), hACE2 (F), hACE2 with N501Y (G), and the alignment of bACE2-Ra with N501Y (H) are shown separately. **I.** The alignment of bACE2-Ra and hACE2 neighboring residue K417. **J & K.** The surface electrostatic potential of predicted E484Q (J) and E484K (K) on bACE2-Ra. The scale is from -50.0 to +50.0 kTe^-1^. **L** The location of L452 on RBD.

To evaluate whether the adaptability of SARS-CoV-2 variants to the intermediate horseshoe bat ACE2 changed, flow cytometry and SPR assays were performed. All SARS-CoV-2 variant RBDs bound to bACE2-Ra and hACE2 **(Fig. 5A and B; Fig. S3A and B; Fig. S4; Table S2)**. The schematic diagrams of mutations in SARS-CoV-2 variant RBDs were shown in the supplementary (**Fig. S5**). Notably, Alpha RBD, containing the N501Y mutation, showed significantly reduced binding to bACE2-Ra. However, N501Y has been reported to enhance contact of SARS-CoV-2 with human, mouse and dog ACE2s 21, 27. In addition, RBDs containing the N501Y mutation (Alpha, Beta, Gamma and Theta) showed stronger binding with hACE2 **(Fig. 5B and D)**. In contrast to Alpha, Beta and Gamma RBDs increased the binding affinity to bACE2-Ra, indicating that other substitutions in these two variants may enhance the binding of RBD to bACE2-Ra to balance the N501Y mutation **(Fig. 5A and C)**.

Structure analysis indicated that in the bACE2-Ra/SARS-CoV-2 RBD complex, K353 of bACE2-Ra formed hydrogen bonds with G496, Q498 and G502 of the SARS-CoV-2 RBD **(Fig. 5E)**. However, K353 of hACE2 was not in contact with Q498 of the RBD in the complex **(Fig. 5F)**, indicating that the interaction network of K353 played a more critical role in bACE2-Ra/SARS-CoV-2 RBD complex than in hACE2/SARS-CoV-2 RBD complex. When N501 of the RBD was substituted with Y501 (Alpha strain), the interaction network with K353 was destroyed. Y501 in the RBD formed a π-π stacking interaction and a hydrogen bond with Y41 and K353 of hACE2, respectively, which enhanced the binding affinity of the RBD with hACE2** (Fig. 5G)**. In the bACE2-Ra/SARS-CoV-2 RBD complex, the N501Y mutation broke the interaction network of K353 and decreased the binding affinity of the RBD to bACE2-Ra **(Fig. 5H)**. As mentioned above, in bACE2-Ra, N31 is uncharged, while E35 is negatively charged. In the SARS-CoV-2 RBD, the negatively-charged E484 repelled E35 in the SARS-CoV-2 RBD. The E484Q substitution (Kappa strain) decreased the negative charge on the binding surface of SARS-CoV-2 RBD and enhanced its binding affinity of SARS-CoV-2 RBD with hACE2 **(Fig. 5J)**. E484K substitution caused the interaction interface in the RBD to change to a positive charge, resulting in an enhancement of the binding affinity with hACE2 **(Fig. 5K)**. K417 in the RBD formed a salt bridge with D30 in hACE2 and bACE2-Ra **(Fig. 5I)**. Combined with substitutions at residues 417, 484 and 501, Beta and Gamma strains enhanced binding with bACE2-Ra **(Fig. 5C)**. Residue 452 in the SARS-CoV-2 RBD was not in contact with bACE2-Ra, therefore, did not influence their binding **(Fig. 5L)**.

## Discussion

Bats are identified as reservoir hosts of CoVs, some of which are closely related to SARS-CoV or SARS-CoV-2 11, 28-32, two important human-infecting CoVs. In addition, substantial evidence indicates that SARS-CoV-2 might originate from bats and be transmitted to humans via intermediate hosts 33-35. RaTG13, one of the closest relatives of SARS-CoV-2, was sequenced from intermediate horseshoe bats 2. We evaluated the binding affinity between bACE2-Ra and SARS-CoV-2 RBD and found that bACE2-Ra could bind to SARS-CoV-2 RBD. However, the binding affinity was lower than that of hACE2, consistent with pseudovirus infection. The complex structure of bACE2-Ra/SARS-CoV-2 RBD revealed the molecular basis for their interaction. Furthermore, it is the second complex structure of bat ACE2 complexed with SARS-CoV-2 RBD, following big-eared horseshoe bat and providing more correlation between horseshoe bats and CoVs 20.

Notably, ACE2 polymorphism in bats influences binding with SARS-related virus RBD 36. bACE2-Ra is also polymorphic, including two main types of bACE2-Ra sequences uploaded on the database. One contains R34 and E38 and was the main target of this study (GenBank: QMQ39222.1, bACE2-Ra). The other is more similar to hACE2, with the combination of H34 and D38 (GenBank: QMQ39227.1, bACE2-HD), which only carries four different residues on the interface compared with hACE2. Interestingly, according to the flow cytometry results, SARS-CoV-2 RBD could bind to both bACE2-Ra and bACE2-HD. However, the binding capacity of RaTG13 RBD was different. RaTG13 RBD could hardly bind to bACE2-Ra, whereas RaTG13 RBD could bind to bACE2-HD much better than that of bACE2-Ra 37. Our previous work reported that the binding affinity of bACE2-Ra and the RaTG13 RBD is 33.2 μM, which could not be manifested by flow cytometry. Compared to the SARS-CoV-2 RBD, the RaTG13 RBD contains six different residues (F449, L486, Y493, Y498, D501 and H505) on the interaction interface. After changed into the corresponding residue on the SARS-CoV-2 RBD, the RaTG13 RBD containing D501N or H505Y substitution increased the binding affinity with bACE2-Ra by ~58-fold or ~6-fold, respectively 12. When all the six different residues in the RaTG13 RBD on the interaction interface were substituted by the consistent amino acids in the RBD, the binding affinity increased ~204-fold. It indicates that D501 and H505 are the key residues for the weak interaction between RaTG13 RBD.

The interspecies transmission of SARS-CoV-2 poses a significant threat to global public health. Once SARS-CoV-2 circulates among bats, the suppressed innate immune response and special adaptive immune system of bats may facilitate the SARS-CoV-2 evolution 38-40. In particular, recombination between different lineages in the same reservoir host has been proved as an important virus evolution method, which might give birth to new variants or even new viruses 41, 42. The World Organization for Animal Health (OIE) has reported the natural infection of SARS-CoV-2 in twenty species, including cats, dogs, minks, otters, ferrets, lions, tigers, pumas, snow leopards, gorillas, white-tailed deer, fishing cats, binturongs, coatimundis, spotted hyenas, Eurasian lynxes, Canada lynxes, hippos, hamsters and mule deer (https://www.woah.org/app/uploads/2022/04/sars-cov-2-situation-report-11.pdf) 43. One of the best-known examples is the two-way transmission between human and minks, that minks had transmitted SARS-CoV-2 strains with an animal sequence signature back to humans and caused further community transmission 44. Thus, it is vital to continuously monitor potential hosts.

Multiple residues in ACE2 orthologs have been reported to influence RBD binding. Among them, Y41 and Q42 are preferred by the SARS-CoV-2 RBD to H41 and E42 20. Other influencing factors include the hydrophobic patch formed by F28, L79, M82, and Y83 of hACE2 and F486 and Y489 of SARS-CoV-2 RBD 16. In this study, we first found that E35 of bACE2-Ra had a sabotaging impact on RBD interactions owing to its electrostatic repulsion on E484. In the SARS-CoV-2 RBD, residues 493, 498 and 501 were identified as mutational hotspots 24. Notably, the N501Y substitution has been reported to be an enhancing substitution when binding to human or mouse ACE2 45-47, while decreasing its binding affinity to bACE2-Ra. Structural analysis showed that the sabotaging effect resulted from the destruction of interaction networks around K353. In addition, residue 484 was found to exert differential influence depending on the electrical charge of its side chain. A similar effect has been observed in the RBD of the pangolin coronavirus GX/P2V/2017 and Omicron variant 46, 48.

Herein, we mainly focus on the molecular mechanism of SARS-CoV-2 RBD to bACE2-Ra. However, receptor-binding is not the only factor determining SARS-CoV-2 entry. There are many other co-factors playing the role in SARS-CoV-2 entry to the cells, such as neuropilin-1, phosphatidylserine receptors, heparan sulfate proteoglycans, etc. 49. The authentic SARS-CoV-2 infection assay in bats needs to be further studied.

In summary, we evaluated the molecular mechanism of SARS-CoV-2 RBD binding to bACE2-Ra. These results broadened our knowledge of the molecular mechanisms of SARS-CoV-2 RBD binding to bACE2-Ra and indicated that long-term surveillance of intermediate horseshoe bats is vital to minimize spillover risk.

## Materials and methods

### Gene cloning

Full-length coding sequences of bACE2-Ra and hACE2 were synthesized and cloned into the pEGFP-N1 vector for flow cytometry analysis. The peptide domain coding sequences of hACE2 (residues 19-615, GenBank: BAJ21180.1) and bACE2-Ra (residues 19-615, GenBank: QMQ39222.1) were synthesized and cloned into the pET-21a vector for protein expression.

The coding sequences of the SARS-CoV-2 NTD (residues 13-304 of Spike protein, GISAID: EPI_ISL_402119), SARS-CoV-2 RBD (residues 319-541, GISAID: EPI_ISL_402119) and variant RBDs (residues 319-541) were cloned into pCAGGS vectors with an N-terminal signal peptide and a C-terminal histidine tag for protein expression. Full-length coding sequences of SARS-CoV-2 Spike (GISAID: EPI_ISL_402119) were cloned into pCAGGS vectors for pseudotyped virus preparation.

### Protein expression and purification

The pET-21a plasmids containing hACE2, bACE2-Ra, mutant hACE2 and mutant bACE2-Ra were transformed into *Escherichia coli* (*E. coli*) strain BL21 (DE3) for protein expression. After induction with 1 mM IPTG for 6 h, ACE2s were overexpressed as inclusion bodies and refolded, as previously reported 50. After refolding, the proteins were concentrated and exchanged in a buffer (20 mM Tris-HCl pH 8.0 and 150 mM NaCl). Proteins were further purified by gel filtration using a HiLoad 16/600 Superdex^TM^ 200 pg column and ÄKTA System (GE Healthcare, Chicago, USA).

Plasmids containing SARS-CoV-2 NTD, SARS-CoV-2 RBD and variant RBDs were transiently transfected into HEK293F cells using PEI. After seven days, the supernatants were collected, filtered and purified using a HisTrap^TM^ HP column (GE Healthcare). Proteins were further purified by gel filtration using a HiLoad 16/600 Superdex^TM^ 200 pg column and ÄKTA System (GE Healthcare). Purified proteins were stored in gel filtration buffer (20 mM Tris-HCl pH 8.0 and 150 mM NaCl).

### Surface plasmon resonance (SPR) analysis

For all measurements, phosphate-buffered saline containing Tween 20 (PBST; 1.8 mM KH_2_PO_4_, 10 mM Na_2_HPO_4_ pH 7.4, 137 mM NaCl, 2.7 mM KCl and 0.05% (v/v) Tween 20) was used as the running buffer. hACE2, bACE2-Ra, mutant hACE2 and mutant bACE2-Ra were transferred into PBST buffer and immobilized on the CM5 chip. SARS-CoV-2-RBD or variant RBDs were serially diluted and flowed through the CM5 chip in PBST buffer. Binding affinities were measured using a BIAcore 8K system (GE Healthcare) at 25℃ in single-cycle mode. After each cycle, the sensor chips were regenerated with glycine (pH 1.7). Kinetics or steady states were analyzed using BIAcore Insight software (GE Healthcare) with a 1:1 Langmuir binding model. Graphics were generated using OriginPro 9.1 software (OriginLab Corporation, Northampton, USA).

### Flow cytometry analysis

Plasmids containing bACE2-Ra or hACE2 sequences and pEGFP-N1 vectors were transiently transfected into Baby Hamster Syrian Kidney (BHK-21) cells using PEI. Cells were collected 24 h after transfection, suspended in PBS (with 0.5% FBS) and incubated with the 10 mg/mL test proteins (SARS-CoV-2 NTD, SARS-CoV-2 RBD and variant RBDs) on ice for 1 h. Subsequently, the cells were washed three times with PBS and further incubated with anti-His/APC antibodies (1:500, Miltenyi Biotec, Bergisch Gladbach, Germany) on ice for 1 h. Finally, the cells were washed thrice and analyzed using BD FACS Canto Flow cytometer (BD Biosciences, Franklin Lakes, USA). The data were processed and generated using the FlowJo 10.6 software (FlowJo LLC, Ashland, USA).

### Pseudotyped virus preparation and infection

SARS-CoV-2 pseudotyped viruses were constructed using a GFP-encoding replication-deficient vesicular stomatitis virus (VSV) vector backbone (VSV-ΔG-GFP). HEK293T cells were transfected with 30 μg of the plasmid for spike protein expression. VSV-ΔG-GFP was added 24 h after transfection. The inoculum was removed after incubating for 1 h at 37 °C. The culture medium was then changed to DMEM supplemented with 10% FBS and 10 μg/mL of anti‐VSV‐G antibody (I1‐Hybridoma ATCC^®^ CRL2700) after washing cells with PBS. The pseudotyped viruses were harvested 20 h after inoculation, filtered, aliquoted and stored at -80°C.

Pseudovirus particles of SARS‐CoV‐2 were added to each well of a 96‐well plate containing HeLa‐bACE2-Ra cells. HeLa cells were used as controls. The plates were imaged at 15 h after transfection. Fluorescent cells were counted using a CQ1 confocal image cytometer (Yokogawa Electric, Tokyo, Japan). Each group contained three replicates.

### Crystallization, data collection and structure determination

Sitting-drop vapor diffusion was used for the complex crystallization. The purified bACE2-Ra/ SARS-CoV-2 RBD complex proteins were concentrated to 5 or 10 mg/mL. Then, 0.8 μL protein was mixed with 0.8 μL reservoir solution. The resulting solution was sealed and equilibrated against 100 µL of the reservoir solution at 4°C and 18°C. Crystals of bACE2-Ra/SARS-CoV-2 RBD complexes were obtained in solution consisting of 0.2 M sodium acetate trihydrate, 0.1 M sodium citrate pH 5.5, 5 % (w/v) PEG 4000 with 10 mg/mL concentration.

Crystals were transferred and soaked in anti-freezing buffer (reservoir solution, 20 % (v/v) glycerol). Crystals were then collected using mini loops and frozen in liquid nitrogen. Diffraction data were collected at Shanghai Synchrotron Radiation Facility (SSRF, Shanghai, China) 19U beamline. The high-resolution structural dataset was processed and scaled using the HKL2000 software package (https://hkl-xray.com/) 51. The original model was determined by molecular replacement, with the reported structure of hACE2/SARS-CoV-2 RBD complex (PDB ID: 6LZG) as reference via the Phaser MR program in CCP4 (http://www.ccp4.ac.uk/) 52. The atomic model was refined using the phenix.refine program in Phenix 52 and manually adjusted using Coot (https://www2.mrc-lmb.cam.ac.uk/personal/pemsley/coot/) 53. The stereochemical qualities of the final model were assessed using MolProbity 54. The data collection, processing and refinement statistics are summarized in Supplementary Table 1. All structural figures were generated using Pymol software (https://pymol.org/2/).

## Supplementary Material

Supplementary figures and tables.

## Figures and Tables

**Table 1:** Comparison of SARS-CoV-2 RBD binding to bACE2Ra, hACE2 and bACE2-Rm

SARS-CoV-2 RBD	bACE2-Ra	hACE2	bACE2-Rm
K417 (6/4/6)	D30 (3,1), R34 (3)	D30 (4,1)	D30 (6,1)
G446 (1/4/4)	Q42 (1)	Q42 (4,1)	E42 (4)
Y449 (12/13/17)	E38 (5), Q42 (7,1)	D38 (9,1), Q42 (4,1)	D38 (9,1), E42 (8,1)
Y453 (13/6/3)	R34 (13,1)	H34 (6)	S34 (3)
L455 (12/14/12)	D30 (2), N31 (2), R34 (8)	D30 (2), K31 (2), H34 (10)	D30 (7), K31 (3), S34 (2)
F456 (18/14/19)	I27 (10), D30 (4),N31 (4)	T27 (5), D30 (4), K31 (5)	K27 (11), D30 (4), K31 (4)
Y473 (0/1/6)	--	T27 (1)	K27 (6,1)
A475 (7/9/10)	S19 (2), R24 (3), I27 (2)	S19 (3,1), Q24 (4), T27 (2)	E24 (8,1), K27 (2)
G476 (2/9/9)	R24 (2)	S19 (4), Q24 (5)	E24 (9)
E484 (0/1/2)	--	K31 (1)	K31 (2,1)
F486 (19/22/17)	R24 (1), L79 (2), N82 (7), Y83 (9)	L79 (2), M82 (9),Y83 (11)	L79 (2), N82 (7), Y83 (8)
N487 (15/23/21)	R24 (10,1), Y83 (5,1)	Q24 (15,1), Y83 (8,1)	E24 (16,1), Y83 (5,1)
Y489 (21/21/25)	I27 (4), F28 (8), N31 (8), Y83 (1,1)	T27 (7), F28 (7), K31 (6), Y83 (1)	K31 (9), F28 (10), Y83 (1,1), K27 (4), E24 (1)
F490 (0/2/3)	--	K31 (2)	K31 (3)
Q493 (9/20/16)	N31 (4,1), E35 (5)	K31 (3), H34 (6), E35 (11,1)	K31 (7), S34 (6), K35 (3)
G496 (16/7/10)	E38 (11,1), K353 (5,1)	D38 (2), K353 (5,1)	D38 (4), K353 (6,1)
Q498 (13/36/16)	E38 (1), Y41 (7), K353 (5,1)	D38 (2), Y41 (17), Q42 (12,1), L45 (5)	D38 (4), Y41 (7), E42 (3), K353 (2)
P499 (0/0/1)	--	--	K330 (1)
T500 (32/27/36)	Y41 (7,2), L45 (2), N330 (9), D355 (10), R357 (4)	Y41 (7,1), L45 (1), N330 (8), D355 (8,1), R357 (3)	Y41 (10,1), L45 (3), K330 (11,1), D355 (9), R357 (3)
N501 (27/19/26)	Y41 (10,1), K353 (17)	Y41 (8,1), K353 (11)	Y41 (14,1), K330 (1), K353 (11)
G502 (13/12/13)	K353 (5,1), G354 (7), D355 (1)	K353 (4,1), G354 (7), D355 (1)	K353 (6,1), G354 (6), D355 (1)
Y505 (36/40/39)	E37 (9,1), K353 (23), G354 (3), R393 (1)	E37 (7), K353 (28), G354 (4), R393 (1)	K353 (27), E37 (8,1), R393 (2), G354 (2)
Total (272/304/311)	272 (15)	304 (14)	311 (15)

“--” represents the residue in the SARS-CoV-2 RBD does not contact with the corresponding residue in the ACE2.
