# Supplementary material for: Structural basis of SARS-CoV-2 and its variants binding to intermediate horseshoe bat ACE2
Source: Int J Biol Sci. 2022 Jul 11;18(12):4658–68. doi: 10.7150/ijbs.73640 (PMC9305271; doi:10.7150/ijbs.73640)
Supplement: Supplementary file 1 — Supplementary figures and tables. [file ijbsv18p4658s1.pdf]

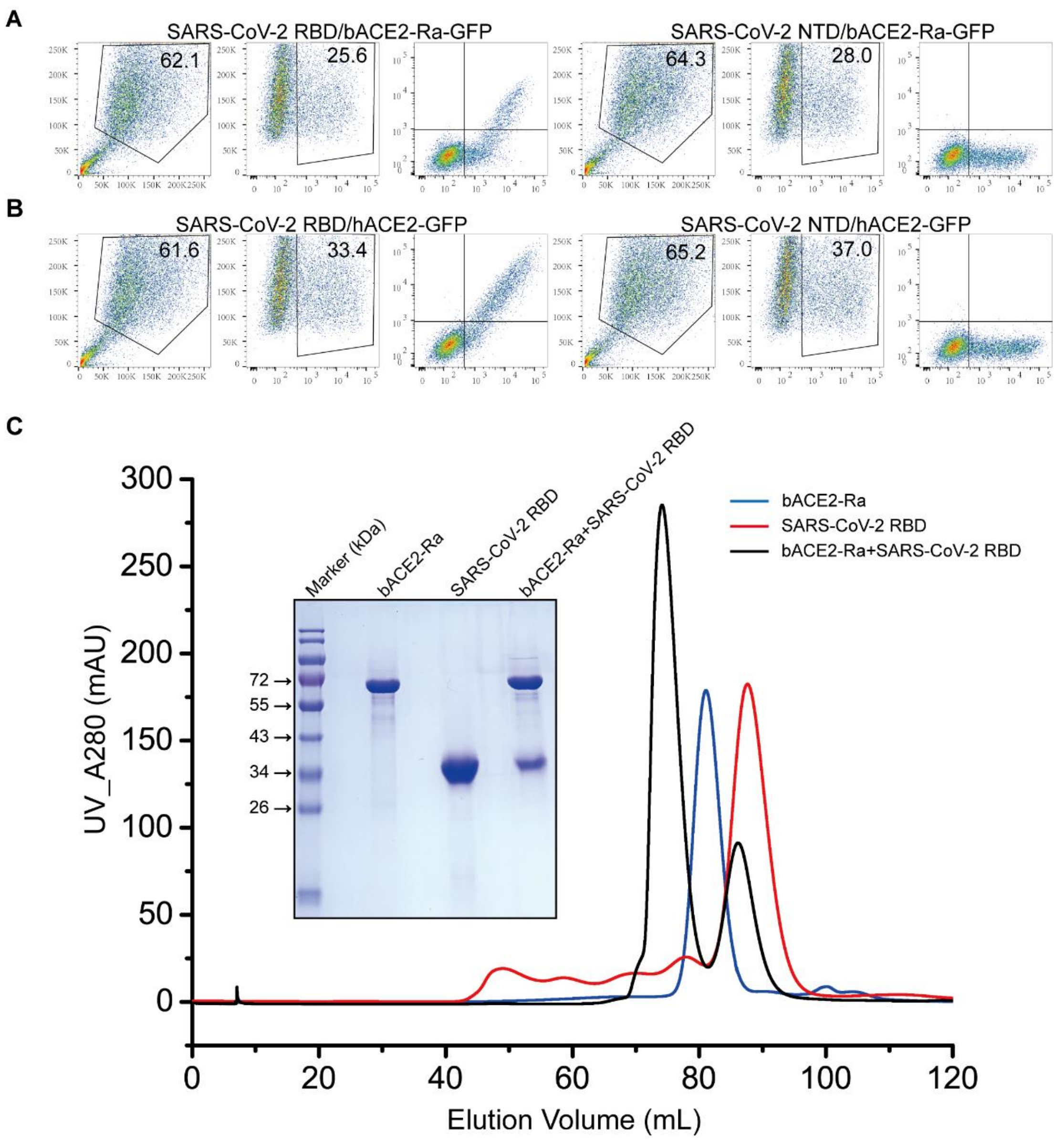

Figure S1. The gating strategy and gel filtration profiles.

A & B. The gating strategy for SARS-CoV-2 RBD binding to BHK-21 cells expressing bACE2-Ra-GFP (A) or hACE2-GFP (B). Cells were initially gated based on the forward scatter (FSC) and the side scatter (SSC) signals. As cells are not visibly clustered, they were subsequently gated based on GFP fluorescent signals. Then APC signals from anti-His antibodies were shown to divide the cells into RBD binding positive clusters and negative clusters. NTD were used as the negative control.

C. Gel filtration profiles of bACE2-Ra, SARS-CoV-2 RBD and the bACE2-Ra/SARS-CoV-2 RBD complex. The separation profiles of each pooled samples on SDS-PAGE were shown in reducing conditions (+DTT).

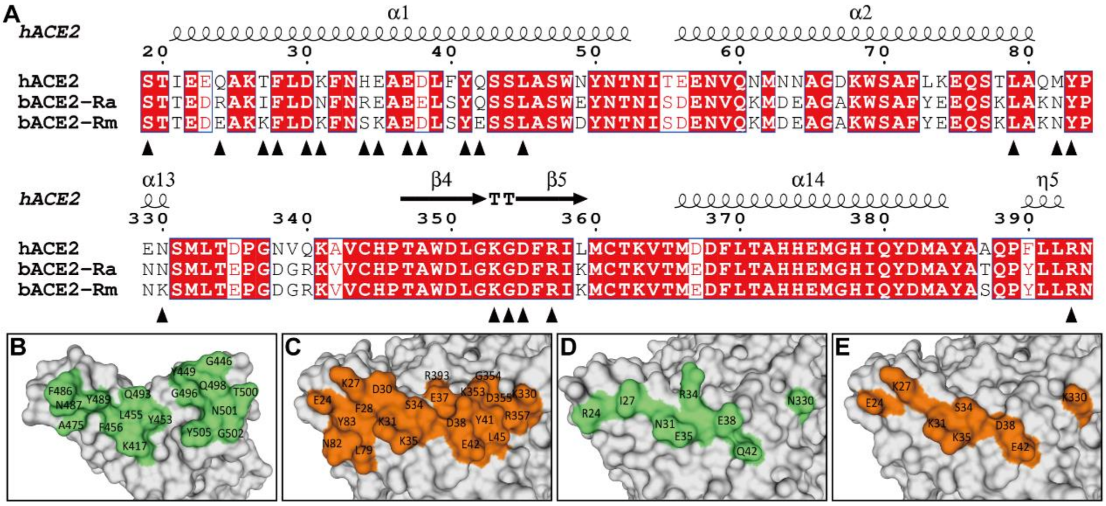

Figure S2. Sequence alignment of critical residues on ACE2s and comparison of the interaction surface.

A. Sequence alignment of the critical residues of bACE2-Ra, hACE2 and bACE2-Rm participating in the interaction. The secondary structure iss depicted on the top referring to the structure of hACE2 (PDB ID: 6LZG).

B. The interaction surface of SARS-CoV-2 RBD when binding to bACE2-Ra.

C–E. The interaction interface of bACE2-Rm (C). Different interacting residues between bACE2-Ra (D) and bACE2-Rm (E) on the interaction interface are shown apart.

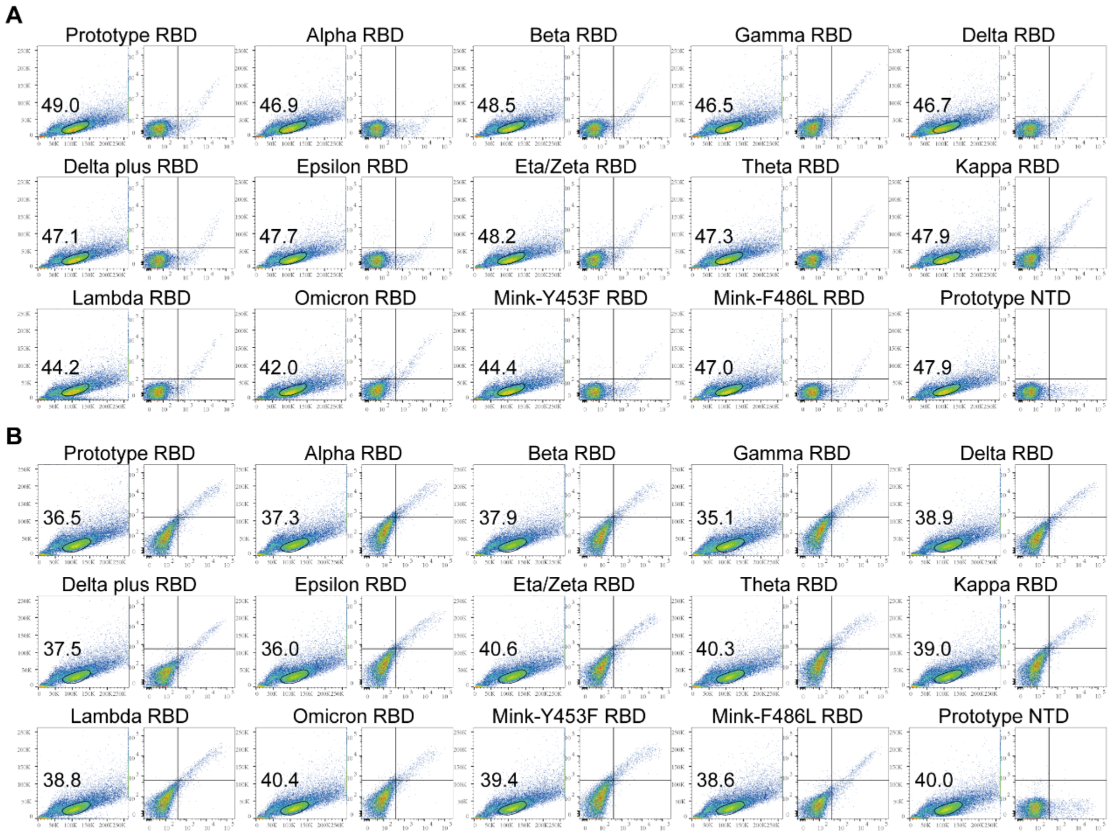

Figure S3. The gating strategy for variant flow cytometry assays.

A & B. The gating strategy for variants RBD binding to BHK-21 cells expressing bACE2-Ra-GFP (C) or hACE2-GFP (D). Cells were initially gated based on FSC and SSC signals, and the cell clusters were evident. Hence, APC signals were shown to divide the cells into variants RBD binding positive clusters and negative clusters. NTD were used as the negative control.

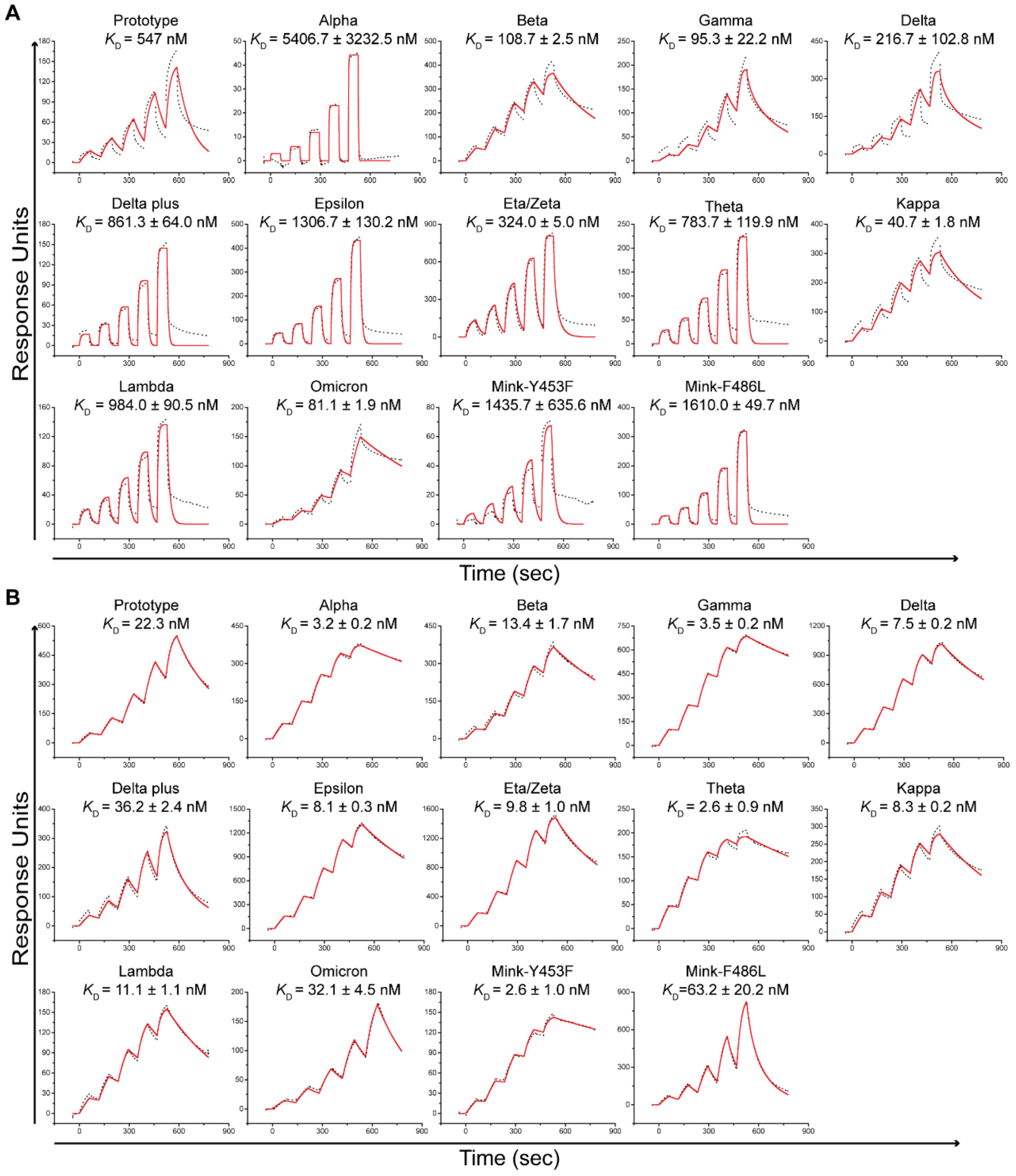

Figure S4. Binding between bACE2-Ra or hACE2 with SARS-CoV-2 variant RBDs.

A & B. Surface plasmon resonance assay depicting the binding affinity of bACE2-Ra (A) or hACE2 (B) with SARS-CoV-2 VOC RBDs. The raw and fitted curves were respectively shown as black dotted lines and red solid lines.

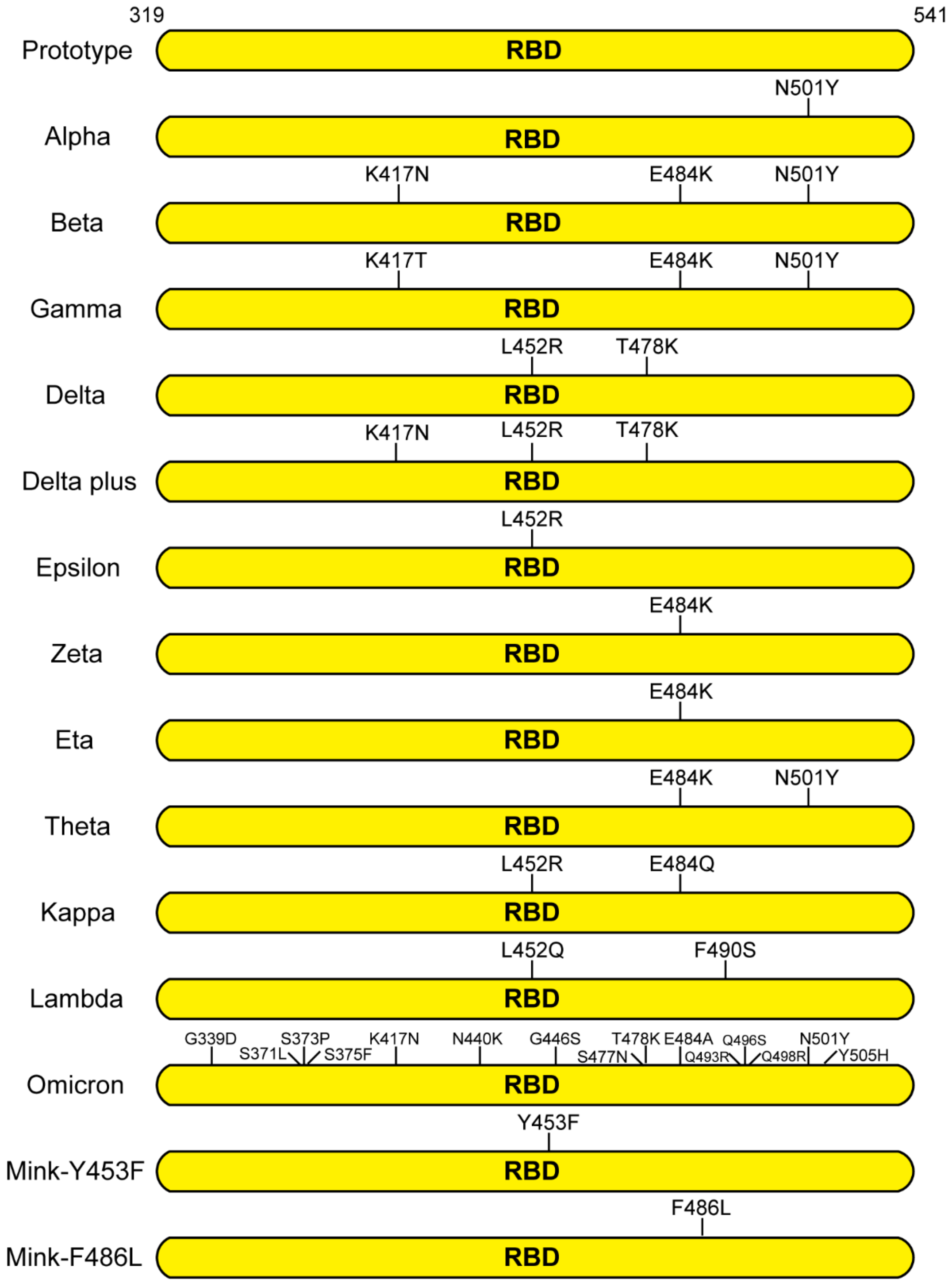

Figure S5. Schematic diagrams of mutations in SARS-CoV-2 variant RBDs.

Table S1. Crystallographic data collection and refinement statistics.

	bACE2-Ra/SARS-CoV-2 RBD (PDB ID: 7XA7)
**Data collection**	
Space group	$P2_12_12_1$
Cell dimensions	
a, b, c (Å)	63.94, 130.85, 564.83
α, β, γ (°)	90.00, 90.00, 90.00
Resolution (Å)	50.00 - 3.30 (3.42 - 3.30)
Unique reflections	69914
Completeness (%)	96.5 (99.2)
R_{merge}	0.218 (1.345)
$I/\sigma I$	8.8 (1.2)
$CC_{1/2}$ (%)	98.9 (60.3)
Redundancy	8.9 (8.3)
**Refinement**	
Resolution (Å)	40.28 - 3.31 (3.43 - 3.31)
No. of reflections	69871 (6847)
R_{work}/R_{free}	0.2294/0.2526
No. of atoms	
Protein	25747
Ligand/ion	60
Water	42
B-factor	
Protein	89.0
Ligand/ion	107.7
Water	51.7
R.M.S. deviations	
Bond length (Å)	0.002
Bond angles (°)	0.520
Ramachandran statistics (%)	
Favored	97.46
Allowed	2.51
Disallowed	0.03

Table S2. Kinetic statistics for SPR assays of variants RBD.

Stationary phase	Mobile phase	Immobilization level (RU)	Concentration (nM)	K_D (M)	K_a ($M^{-1}\cdot s^{-1}$)	K_d (s^{-1})
Intermediate horseshoe bat ACE2	Prototype RBD	9379.2	800, 400, 200, 100, 50	5.47×10^{-7}	2.80×10^{4}	1.53×10^{-2}
Intermediate horseshoe bat ACE2	Alpha RBD	9379.2	800, 400, 200, 100, 50	$5.41\times10^{-6} \pm 3.23\times10^{-6}$	$2.06\times10^{5} \pm 1.88\times10^{5}$	$5.54\times10^{-1} \pm 5.55\times10^{-2}$
Intermediate horseshoe bat ACE2	Beta RBD	9379.2	800, 400, 200, 100, 50	$1.08\times10^{-7} \pm 24.9\times10^{-8}$	$5.30\times10^{5} \pm 2.05\times10^{5}$	$5.76\times10^{-2} \pm 2.21\times10^{-2}$
Intermediate horseshoe bat ACE2	Gamma RBD	9379.2	800, 400, 200, 100, 50	$9.53\times10^{-8} \pm 2.22\times10^{-8}$	$1.38\times10^{5} \pm 2.47\times10^{4}$	$1.27\times10^{-2} \pm 8.97\times10^{-4}$
Intermediate horseshoe bat ACE2	Delta RBD	9379.2	800, 400, 200, 100, 50	$2.17\times10^{-7} \pm 1.03\times10^{-7}$	$1.85\times10^{4} \pm 1.67\times10^{3}$	$4.17\times10^{-3} \pm 2.38\times10^{-3}$
Intermediate horseshoe bat ACE2	Delta plus RBD	9379.2	800, 400, 200, 100, 50	$8.61\times10^{-7} \pm 6.40\times10^{-8}$	$1.73\times10^{5} \pm 9.11\times10^{3}$	$1.48\times10^{-1} \pm 3.85\times10^{-3}$
Intermediate horseshoe bat ACE2	Epsilon RBD	9379.2	800, 400, 200, 100, 50	$1.31\times10^{-6} \pm 1.30\times10^{-7}$	$8.82\times10^{4} \pm 9.77\times10^{3}$	$1.14\times10^{-1} \pm 9.98\times10^{-4}$
Intermediate horseshoe bat ACE2	Eta/Zeta RBD	9379.2	800, 400, 200, 100, 50	$3.24\times10^{-7} \pm 4.97\times10^{-9}$	$1.29\times10^{6} \pm 2.51\times10^{5}$	$4.17\times10^{-1} \pm 7.61\times10^{-2}$
Intermediate horseshoe bat ACE2	Theta RBD	9379.2	800, 400, 200, 100, 50	$7.84\times10^{-7} \pm 1.20\times10^{-7}$	$1.51\times10^{5} \pm 6.77\times10^{3}$	$1.17\times10^{-1} \pm 1.25\times10^{-2}$
Intermediate horseshoe bat ACE2	Kappa RBD	9379.2	800, 400, 200, 100, 50	$4.00\times10^{-8} \pm 1.82\times10^{-9}$	$4.67\times10^{4} \pm 2.24\times10^{3}$	$1.90\times10^{-3} \pm 9.97\times10^{-5}$
Intermediate horseshoe bat ACE2	Lambda RBD	9379.2	800, 400, 200, 100, 50	$9.84\times10^{-7} \pm 9.04\times10^{-8}$	$1.61\times10^{5} \pm 1.06\times10^{4}$	$1.57\times10^{-1} \pm 4.28\times10^{-3}$
Intermediate horseshoe bat ACE2	Omicron RBD	9379.2	800, 400, 200, 100, 50	$8.11\times10^{-8} \pm 1.91\times10^{-8}$	$2.36\times10^{4} \pm 2.60\times10^{3}$	$1.86\times10^{-3} \pm 1.99\times10^{-4}$
Intermediate horseshoe bat ACE2	Mink-Y453F RBD	9379.2	800, 400, 200, 100, 50	$1.44\times10^{-6} \pm 6.36\times10^{-7}$	$6.21\times10^{4} \pm 1.46\times10^{4}$	$8.75\times10^{-2} \pm 4.12\times10^{-2}$
Intermediate horseshoe bat ACE2	Mink-F486L RBD	9379.2	800, 400, 200, 100, 50	$1.61\times10^{-6} \pm 4.97\times10^{-8}$	$8.09\times10^{4} \pm 2.82\times10^{3}$	$1.30\times10^{-1} \pm 4.20\times10^{-3}$
Human ACE2	Prototype RBD	15746.3	200, 100, 50, 25, 12.5	2.23×10^{-8}	2.56×10^{5}	5.69×10^{-3}
Human ACE2	Alpha RBD	15746.3	200, 100, 50, 25, 12.5	$3.17\times10^{-9} \pm 2.29\times10^{-10}$	$2.38\times10^{5} \pm 4.09\times10^{3}$	$7.56\times10^{-4} \pm 6.44\times10^{-5}$
Human ACE2	Beta RBD	15746.3	200, 100, 50, 25, 12.5	$1.34\times10^{-8} \pm 1.71\times10^{-9}$	$1.41\times10^{5} \pm 7.18\times10^{3}$	$1.88\times10^{-3} \pm 1.43\times10^{-4}$
Human ACE2	Gamma RBD	15746.3	200, 100, 50, 25, 12.5	$3.47\times10^{-9} \pm 1.98\times10^{-10}$	$2.23\times10^{5} \pm 4.26\times10^{3}$	$7.74\times10^{-4} \pm 3.14\times10^{-5}$
Human ACE2	Delta RBD	15746.3	200, 100, 50, 25, 12.5	$7.48\times10^{-9} \pm 2.38\times10^{-10}$	$2.81\times10^{5} \pm 3.18\times10^{4}$	$2.11\times10^{-3} \pm 3.09\times10^{-4}$
Human ACE2	Delta plus RBD	15746.3	200, 100, 50, 25, 12.5	$3.62\times10^{-8} \pm 2.44\times10^{-9}$	$3.17\times10^{5} \pm 9.51\times10^{3}$	$1.15\times10^{-2} \pm 1.10\times10^{-3}$
Human ACE2	Epsilon RBD	15746.3	200, 100, 50, 25, 12.5	$8.05\times10^{-9} \pm 3.35\times10^{-10}$	$2.31\times10^{5} \pm 2.71\times10^{4}$	$1.86\times10^{-3} \pm 2.97\times10^{-4}$
Human ACE2	Eta/Zeta RBD	15746.3	200, 100, 50, 25, 12.5	$9.81\times10^{-9} \pm 9.81\times10^{-10}$	$3.39\times10^{5} \pm 4.54\times10^{4}$	$3.37\times10^{-3} \pm 8.01\times10^{-4}$
Human ACE2	Theta RBD	15746.3	200, 100, 50, 25, 12.5	$2.57\times10^{-9} \pm 8.75\times10^{-10}$	$3.95\times10^{5} \pm 3.02\times10^{4}$	$1.04\times10^{-3} \pm 4.29\times10^{-4}$
Human ACE2	Kappa RBD	15746.3	200, 100, 50, 25, 12.5	$8.34\times10^{-9} \pm 1.94\times10^{-10}$	$2.67\times10^{5} \pm 2.03\times10^{4}$	$2.23\times10^{-3} \pm 1.94\times10^{-4}$
Human ACE2	Lambda RBD	15746.3	200, 100, 50, 25, 12.5	$1.11\times10^{-8} \pm 1.13\times10^{-9}$	$2.06\times10^{5} \pm 1.69\times10^{4}$	$2.28\times10^{-3} \pm 1.48\times10^{-4}$
Human ACE2	Omicron RBD	15746.3	200, 100, 50, 25, 12.5	$3.21\times10^{-8} \pm 4.50\times10^{-9}$	$1.58\times10^{5} \pm 1.26\times10^{4}$	$5.03\times10^{-3} \pm 3.13\times10^{-4}$
Human ACE2	Mink-Y453F RBD	15746.3	200, 100, 50, 25, 12.5	$2.57\times10^{-9} \pm 9.70\times10^{-10}$	$1.90\times10^{5} \pm 9.90\times10^{3}$	$4.61\times10^{-4} \pm 1.48\times10^{-4}$
Human ACE2	Mink-F486L RBD	15746.3	200, 100, 50, 25, 12.5	$6.32\times10^{-8} \pm 2.02\times10^{-8}$	$4.17\times10^{5} \pm 3.06\times10^{4}$	$2.63\times10^{-2} \pm 8.69\times10^{-3}$
